# Stem cell transplantation

**DOI:** 10.1097/HS9.0000000000000266

**Published:** 2019-06-30

**Authors:** Nicolaas Schaap

**Affiliations:** Radboud University Medical Centre Nijmegen, The Netherlands

**Learning goals**

- Preparedness for possible nuclear terrorism is important and possible, multiple parts for preparedness are in place.
- The current state of acute radiation syndrome (ARS) does not substantially differ from the management of pancytopenia in other settings, such as after treatment with myelosuppressive chemotherapy.
- HSCT patients have a high likelihood of severe dysbiosis early after transplantation which is mediated by broad spectrum antibiotics and GvHD itself while real decontamination is rarely achieved.
- Microbiota modulation offers a new option for treatment or even prophylaxis of GvHD.
- The number of patients transplanted using Haplo-HSCT is increasing consistently in Europe and United States.
- Haplo-HSCT with the use of PTCy for GVHD prophylaxis, allows low incidence of grade III to IV acute GVHD, chronic GVHD, and comparable survival with HLA-matched unrelated and cord blood transplantation.

## Introduction

Worldwide radiation accidents are rare, however we learned from the past that these events ask for serious planning in the health care system.. Nuclear accidents in the Chernobyl (1986) and Fukushima (2011) power plants demonstrated that we should draw the attention of the real risk of radiation accidents to the health care. In his presentation Dr Chao will elaborate on the acute radiation syndrome (ARS) in humans. Efficient triage to identify victims without a lethal dose of irradiation who can be rescued is obligatory. On the other hand, the current management of ARS does not differ a lot from the management of pancytopenia in other settings. Extensive triage algorithms and appropriate standardized operation procedures are currently available to improve survival in these victims.

Prof E Holler will focus in his lecture on new insights concerning the changes in the microbiome by using antibiotic prophylaxis that are regularly applied in SCT to prevent infectious complications and GVHD. In contrast, using these antibiotics we induce a massive loss of diversity (dysbiosis) which was associated with an increased risk of GVHD and late transplant related mortality. New molecular sequencing techniques demonstrate not only the loss but also the skewing of the microbiome towards an abundance of single pathogenic bacteria, especially anaerobic species. Fecal microbiota transfer (FMT) may play an important role to restore the diversity of the microbiome.

The recent introduction of new conditioning regimens and graft manipulation techniques have boosted the implementation of haplo-identical stem cell transplantation and offers better chances of survival in this patient category. Dr Ruggeri will talk in her presentation about the two most frequent used conditioning regimens that changed the world of haplo-identical stem cell transplantation, based either on the use of post-transplant cyclophosphamide (PTCy) or on anti-thymocyte globulin (ATG). She will also discuss different stem cell sources (bone marrow vs PBSC), the intensity of the conditioning regimens (RIC vs MAC), new complications and challenges we have to deal with.

